# Coupling spatial analysis and economic valuation of ecosystem services to inform the management of an UNESCO World Biosphere Reserve

**DOI:** 10.1371/journal.pone.0205935

**Published:** 2018-11-05

**Authors:** Charlène Kermagoret, Jérôme Dupras

**Affiliations:** Département des sciences naturelles, Université du Québec en Outaouais, Ripon, Québec, Canada; Swedish University of Agricultural Sciences and Swedish Institute for the Marine Environment, University of Gothenburg, SWEDEN

## 1. Introduction

Man And Biosphere (MAB) program, created in 1971 by UNESCO, has played a pioneering role in supporting and implementing interdisciplinary research to address the challenges of conservation and sustainable use of natural resources from local to global scales [1]. Its main tool, i.e. the World Network of Biosphere Reserves, are ‘science-for-sustainability’ support sites implying the collaboration with a suitable range of actors including local communities and scientists [2]. In 2017, 669 biosphere reserves are listed worldwide, distributed in 120 countries, including 20 transboundary sites. The context in which biosphere reserves operate has greatly changed since the MAB creation but remains highly focused on interdisciplinarity [3]. Ecosystem Services (ES), commonly defined as the contributions of ecosystem structure and function to human well-being [4], has emerged as a key concept for managing the Biosphere Reserves [5]. Indeed, ES are part of the Action Plan for Biosphere Reserves since 2008 through the mission of “maintaining and developing ecological and cultural diversity and securing ecosystem services for human wellbeing” [6] and have been recently reinforced with the targeted outcome (A7) of the Lima Action Plan entitled “Biosphere Reserves recognized as sources and stewards of ES”, declined in three actions—A7.1. Identify ecosystem services and facilitate their long-term provision, including those contributing to health and wellbeing; A7.2. Implement mechanisms for the equitable payment for ecosystem services (PES); A7.3. Implement programmes to preserve, maintain and promote species and varieties of economic and/or cultural value and that underpin the provision of ecosystem services [7].

Born from American ecological studies in the early 70s [8–10], ES have been mainly popularized within the international scientific community after the publications of Costanza [11] and Daily [12] and have been institutionalized through major international initiatives and programs like the Millennium Ecosystem Assessment [4], The Economics of Ecosystems and Biodiversity [13] or more recently, the Intergovernmental Science-Policy Platform on Biodiversity and Ecosystem Services (IPBES) [14]. ES have thus gradually emerged as a major concept in the field of environmental management worldwide with the aim to better include ecosystems in the processes of decision making by taking into account the dependence of human societies on ecosystems [15–16]. ES assessments are carried out from local to global levels [17–19] and are generally used for decisive, technical or informative purposes [20]. Scientific developments, both conceptual and methodological, are constantly reinforcing the ES approach in order to enhance its effectiveness in decision-making. For example, the Ecosystem Services Partnership (ESP), the IBPES, Future Earth or ESMERALDA are programs and initiatives recently established to assess ES in a more comprehensively manner in order to promote the practical application of research findings. MAB Biosphere Reserves take advantage of the cooperation and coordination with these initiatives and programmes, sharing information and experiences in order to contribute to knowledge generation and improve their management [1].

This scientific development leads to advocate the use of integrative methods like mapping, modelling and participatory approaches to assess ES taking into account both supply and demand dimensions of ES [21]. The supply refers to the capacity of ecosystems to provide sustainable services. This biophysical capacity results from the ecological state and functioning and can be assimilated to a potential ES, which will really be expressed if ultimately used [22]. Demand refers to the values that individuals or groups of individuals assign to biodiversity and justifies to consider biodiversity as something to be assessed, protected, and in which to invest [22]. In practice, supply assessment are made by ecologists focusing on the ecosystem characteristics and processes involved in the ES production [23–25] while demand assessment are made by economists exploring different ways to determine the value of services to society [26–28]. In practice, these two types of assessments are relatively disconnected and few empirical studies analyse the relationship between ES supply and demand [29]. In a decision support perspective, the use of Total Economic Value (TEV) is still widespread because it provides an overview of the main services and a single aggregated value that reflects the contribution of various services provided by an ecosystem [30]. TEV expresses in monetary terms the quantity of benefits derived from an ecosystem and can easily be used in cost-benefit models [31]. On the one hand, it is attractive because it differentiates and includes both use values and non-use values of ecosystems [32,13]. On the other hand, criticisms have been raised regarding TEV framework. Indeed, in addition to the methodological biases inherent to their evaluation, non-use values raise the question of the relevance of their evaluation in monetary terms and the question of their commensurability with use values [33].

This study is carried out in the Manicouagan-Uapishka World Biosphere Reserve (MUWBR) and aims to assess both ES supply and demand through a spatial analysis coupled with an economic valuation. A strategic approach is conducted in order to complete the operational command of the reserve team implying time and budget constraints which involves to base the assessment on pre-existing data. Indeed, this work was undertaken initially in the context of an ES monitoring exercise that the MAB program requested of every Biosphere Reserves in order to evaluate the evolution of the reserves.

The Materials and Methods section of this paper offers a more detailed presentation of the case study, describes the selection process of the key ES and indicators to be assessed, and detailed the methodological options used for the ES assessment. The Results section presents the spatial analysis of the ES supply and the economic valuation of the ES annual flow provided by the MUWBR. The final section discusses the challenges and opportunities highlighted through this specific assessment and beyond the case study, the discussion provide some operational perspectives.

## 2. Materials and methods

### 2.1. The Manicouagan-Uapishka World Biosphere Reserve (MUWBR)

**Fig 1:**
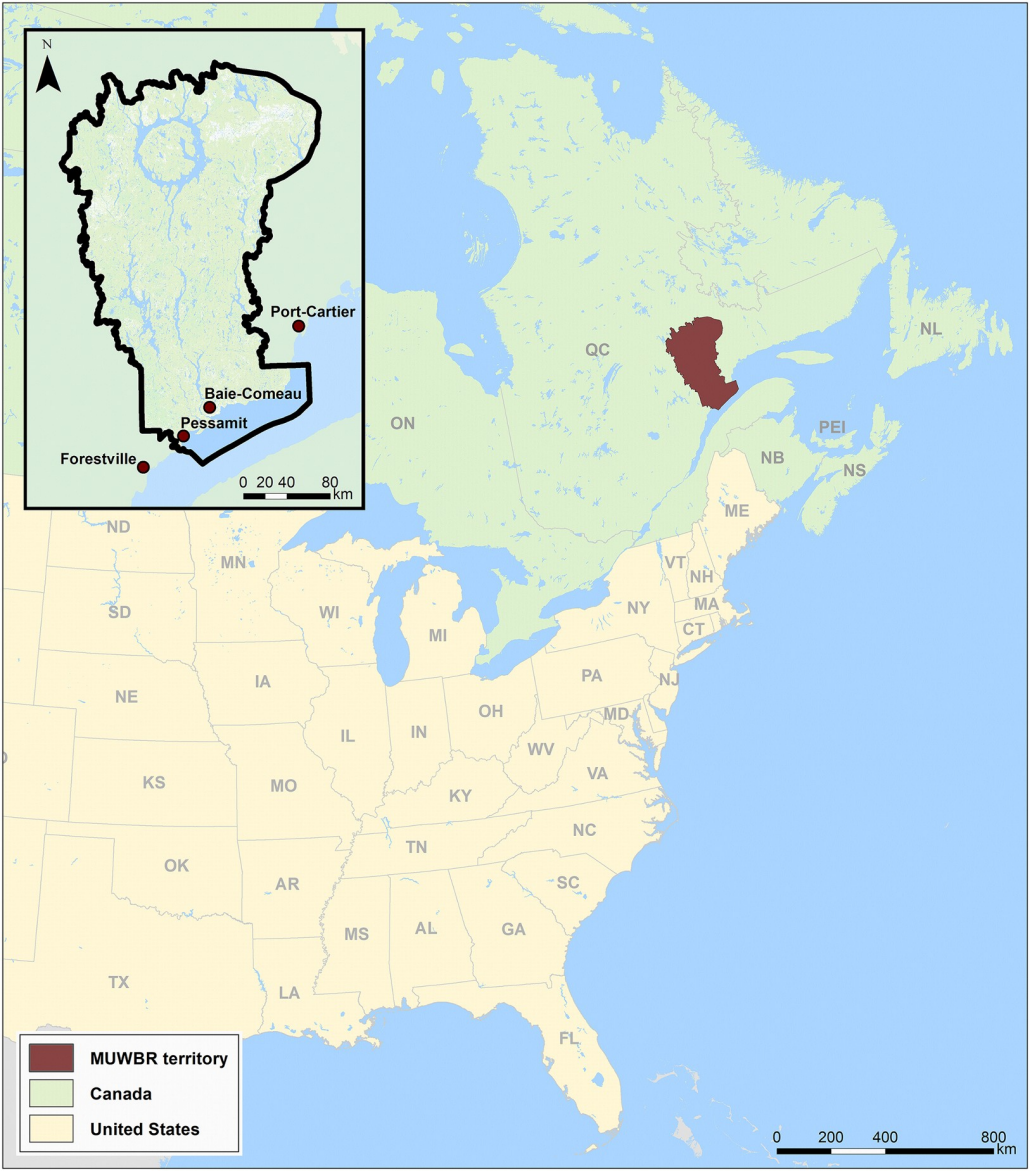
Localisation of the MUWBR.

Created in 2007, the MUWBR is one of the largest World Biosphere Reserve on the planet covering an area of 54 800 km^2^, limited by the Saint Lawrence River to the south and the Manicouagan reservoir to the north (Fig 1).

The MUWBR includes varied ecosystems hosting several species of interest. It contains several remarkable elements at the natural, social and landscape levels. For Manicouagan-Uapishka, the potential for demonstrating sustainable development is at the heart of the Committee’s vision and motivation to work together and to contribute to the Millennium Development Goals. The Manicouagan-Uapishka territory is heavily exploited for its natural resources and the committee wants to make this territory an integrating model of the social, economic and environmental dimensions. It has been working with all sectors of the region for many years to include elements of sustainable development in regional planning [34].

### 2.2. Selection of key ES

Because it is a hotspot of biodiversity [35] and supports different types of activities, MUWBR can provide multiple ES offering benefits at local scales (e.g. flood regulation, recreation) to global scales (e.g. timber, climate regulation). Key ES have been selected by a literature review of the existing international classifications [4,13,36] and a focus group held with academic experts of the forest ecosystem services in northern Quebec. Key ES are defined as those ES that are both significant and relevant for the case study, and for which ecological or economic data are available and can be mobilized within the constraints of time and budget [37]. Some adaptations were made to establish the final list of ES on the basis of pre-existing ES studies in Quebec [38–40]. These adaptations reflect the characteristics of the target site, anticipate the use of transfer value, and aim to avoid double counting. This reclassification results in a selection of 7 key ES for the MUWBR: timber, human food from agriculture, freshwater for energy production, global climate regulation (sequestration and storage of greenhouse gases), habitat provision for biodiversity, recreation and tourism, cultural heritage and cultural diversity i.e. values that humans place on the maintenance of historically important landscapes and forms of land use.

### 2.3. Methods for ES assessment

**Fig 2:**
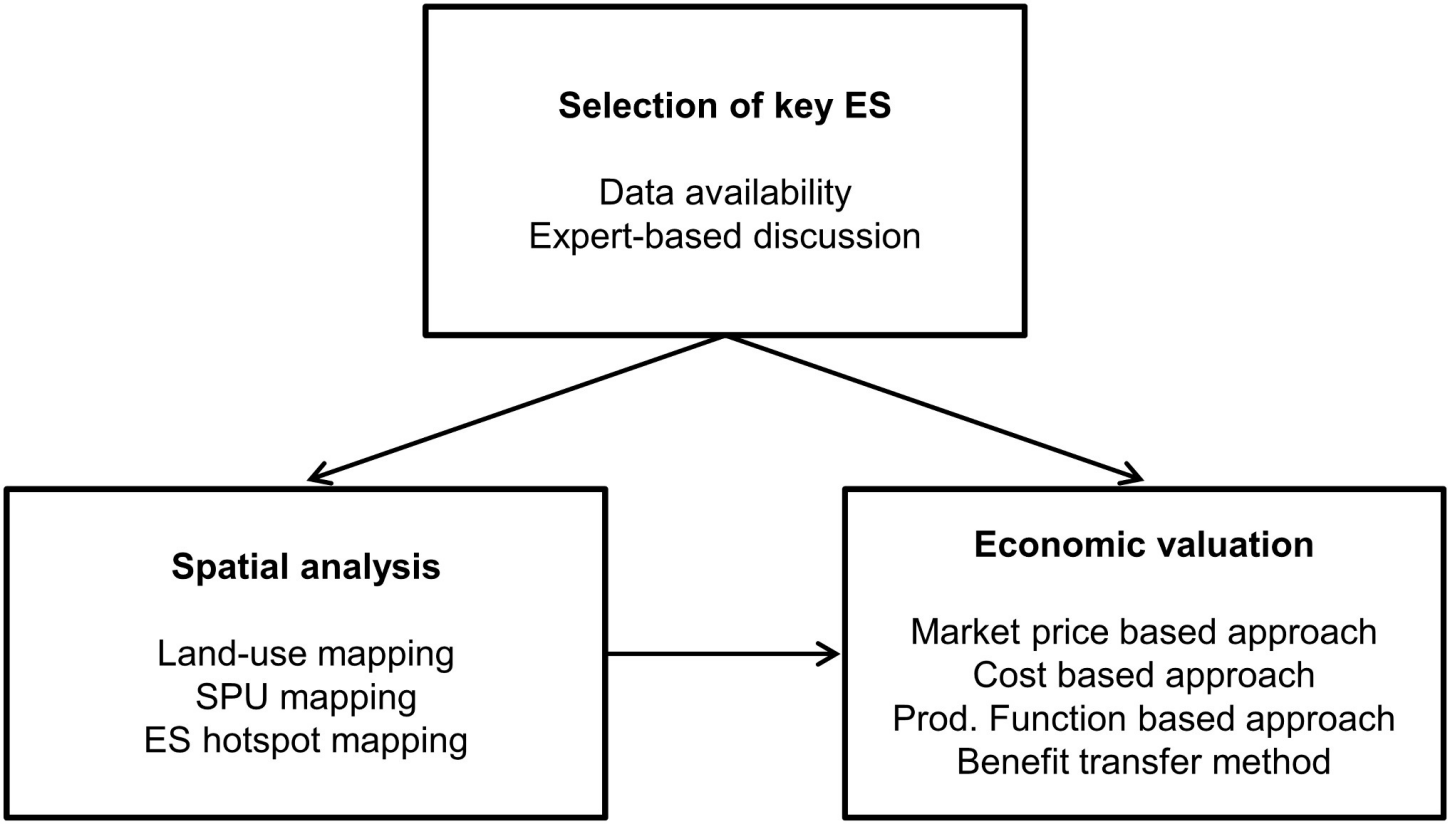
Methodology for the ES assessment.

ES supply and demand are both assessed using an articulation between spatial analysis and economic valuation (Fig 2). On the one hand, a spatial analysis is conducted in order to identify ecosystem Service Providing Unit (SPU) area. SPU area is defined as the spatial unit that is the source of an ES [41] and is considered as one of the most important concept regarding ES supply [42]. On the other hand, an economic valuation is conducted in order to understand how much the ecosystems of the MUWBR contribute to local and regional economic activities and society.

#### 2.3.1. Spatial analysis

Spatial analysis is realised through ArcGIS 10.3 software and is based primarily on three cartographic databases [43–45], as well as on layers provided by the MUWBR team (i.e. reserve boundaries, protected areas, controlled exploited areas and outfitters, localisation of hydroelectric power stations).

Firstly, each key ES is associated with the one or more particular land use classes of the Quebec Land Cover Mapping which are involved in its production. The classes are then grouped into broader land use categories. This clustering allows a better reading of the land uses which are then mapped.

Secondly, based on these clustered categories, SPU areas are mapped and calculated for each ES. SPU is the combination of all the land use categories involved in the its production. It allows to study the distribution of the ES supply in the MUWBR. Calculation of the SPU (ha) also provides a weighting factor which will be useful for the economic valuation.

Finally, an overlay map of the 7 SPU areas is created and informs us about the existence and location of important ES hotspots in the MUWBR defined as small areas that provide multiple key ES.

#### 2.3.2. Economic valuation

As mentioned above, the TEV is usually invoked for economic ES assessment. TEV of ecosystems is classically divided between values of uses and non-uses [46–47]. Use values are the benefits derived from the asset consumption by an agent and from practices related to assets but not involving their consumption (i.e. consumptive and non-consumptive uses) whereas non-use value are the benefits derived by others, provided that the agent’s utility function incorporate ethical or altruistic preferences [48]. The construction of the TEV implies an aggregation of these different values. However, the experts involved in the valuation process favoured use values by considering that the methods of estimating non-use values are less robust and their legitimacy more contested [49]. Use values are estimated through direct market valuation, which has the advantage of using data from actual markets to reflect actual preferences or costs to individuals [50].

Three specific methods are used here:

- Market price-based approaches are used to obtain the value of provisioning services whose products are usually sold in an existing market;
- Cost-based approaches are based on estimations of the costs that would be incurred if ES benefits didn’t existed or needed to be restored or replaced through artificial means;
- Production function-based approaches estimate how much a given ecosystem service contributes to the enhancement of income or productivity.

The benefit transfer method is used when data from actual markets doesn’t exist for the study site. This method allows ES valuation by transferring an existing service valuation estimate from a site with similar characteristics and a similar context [50]. In this study, only values based on similar ecosystems in Quebec through direct market valuation methods are used for benefit transfer.

Monetary values are identified and estimated for 5 of the 7 key ES. Indeed, habitat provision for biodiversity and cultural heritage and diversity are not related to use values so no relevant economic indicators have been identified. The valuation methods used for each ES are presented in Table 1.

**Table 1:** Methods and indicators used for the ES valuation.

ES	Indicator	Market price	Cost	Prod. function	Use of benefit transfer
**Timber**	Timber value	x			No
**Food from agriculture**	Yield	x		x	Yes
**Freshwater for energy production**	Hydroelectric Power			x	Yes
**Global climate regulation**	Social cost of carbon		x		Yes
**Recreation and tourism**	Moose hunting			x	Yes

Finally, the total use value for the MUWBR is calculated and informs us about the relative contribution of each ES to the annual flow of services provided by the reserve.

## 3. Results

### 3.1. Spatial analysis

**Fig 3:**
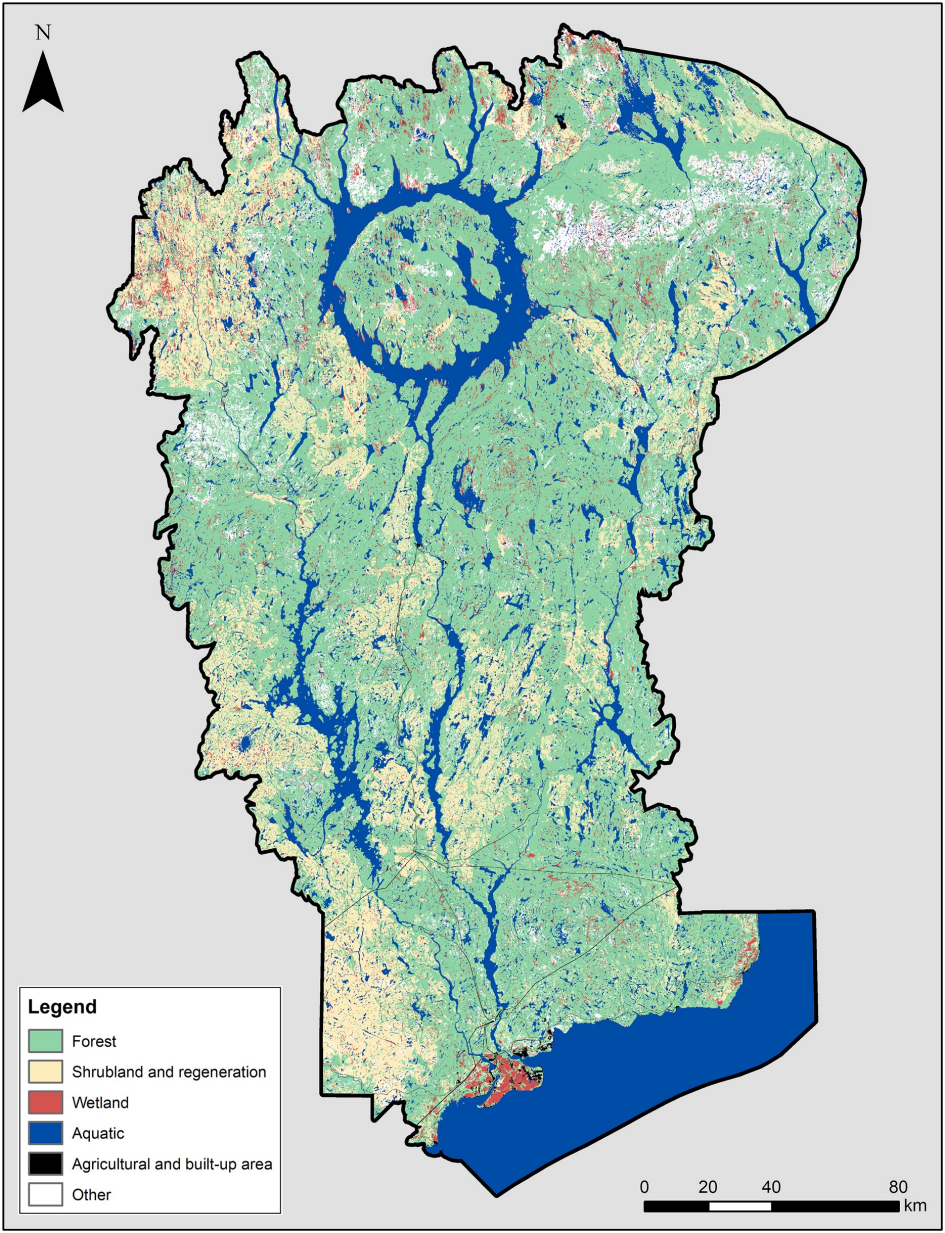
Distribution of the main land use classes in the MUWBR.

A total of 89 land use classes are listed within the MUWBR, and are then grouped into 9 main classes (Table 2). The reserve is well preserved from permanent human occupation since constructed areas and agriculture represent only 0.3%. Forests are the dominant ecosystem, covering 70% of the territory, with conifers constituting the main type of forest cover (46.3%). Aquatic environments (rivers, lakes, tanks, reservoirs) are also very present (ubiquitous), occupying 20% of the territory. Wetlands (marshes, swamps, bogs) represent only 4% of the territory but are located throughout the reserve (Fig 3).

**Table 2:** Main land use classes in the MUWBR (sources: [44–45,51]).

Land uses	Surface Area (Ha)	Percent Cover (%)	Related key ES						
			FA	T	FEP	GCR	HB	RT	CHD
*Agriculture*	2 109	0,0%	x				x		x
*Built-up areas*	18 017	0,3%							
*Shrublands*	202 858	3,7%					x	x	x
*Cuts and regenerations*	664 902	12,3%		x		x	x	x	x
*Coniferous forest*	2 510 543	46,3%		x		x	x	x	x
*Broadleaf or mixed forests*	403 551	7,4%		x		x	x	x	x
*Aquatic environments*	1 021 541	18,8%		x	x	x	x	x	x
*Wetlands*	228 554	4,2%				x	x	x	x
*Other*	368 459	6,8%							

FA: food from agriculture; T: timber; FEP: freshwater for energy production; GCR: global climate regulation; HB: habitat provision for biodiversity; RT: recreation and tourism; CHD: cultural heritage and diversity.

**Fig 4:**
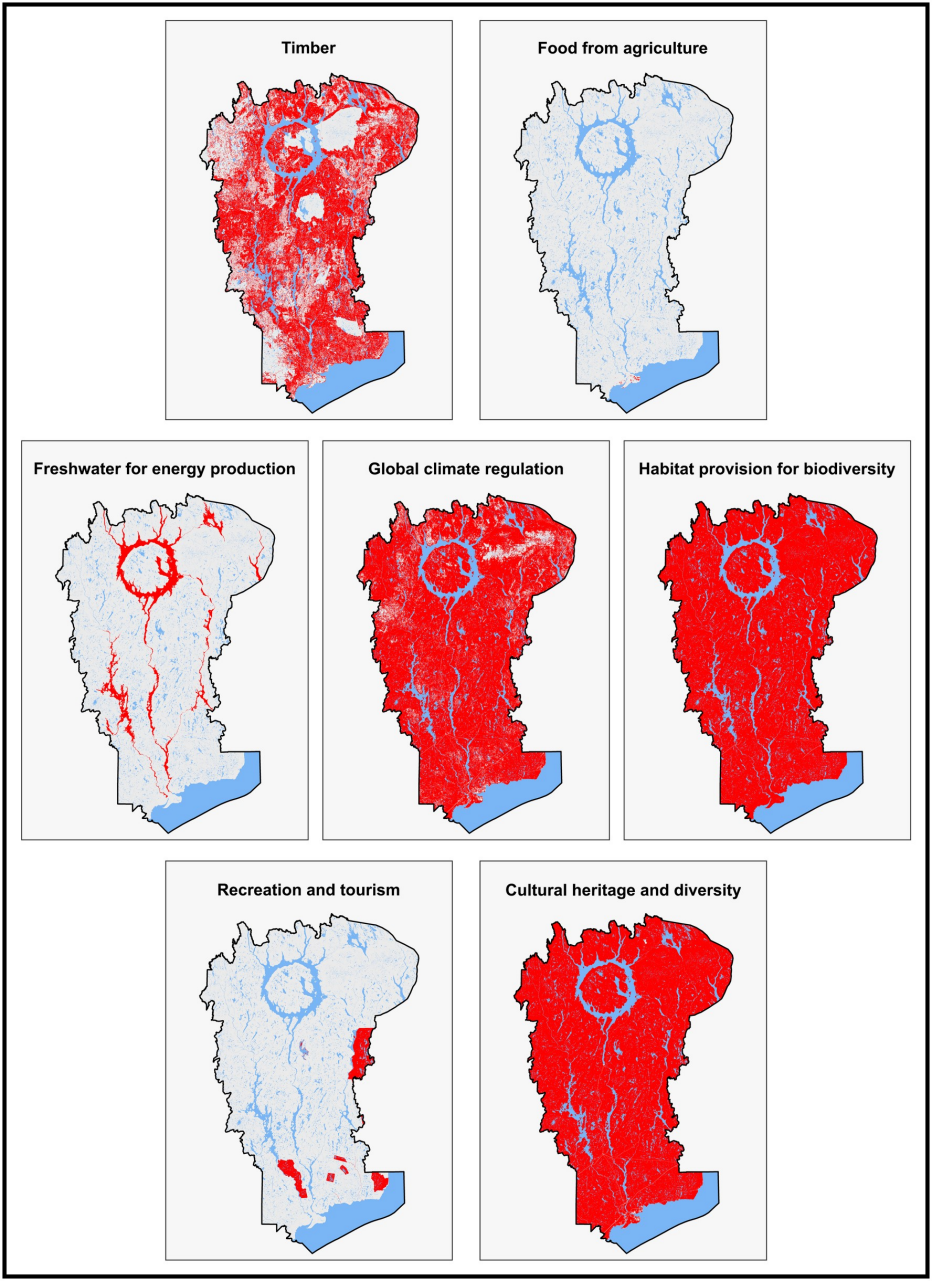
Location of the SPU for each ES.

**Fig 5:**
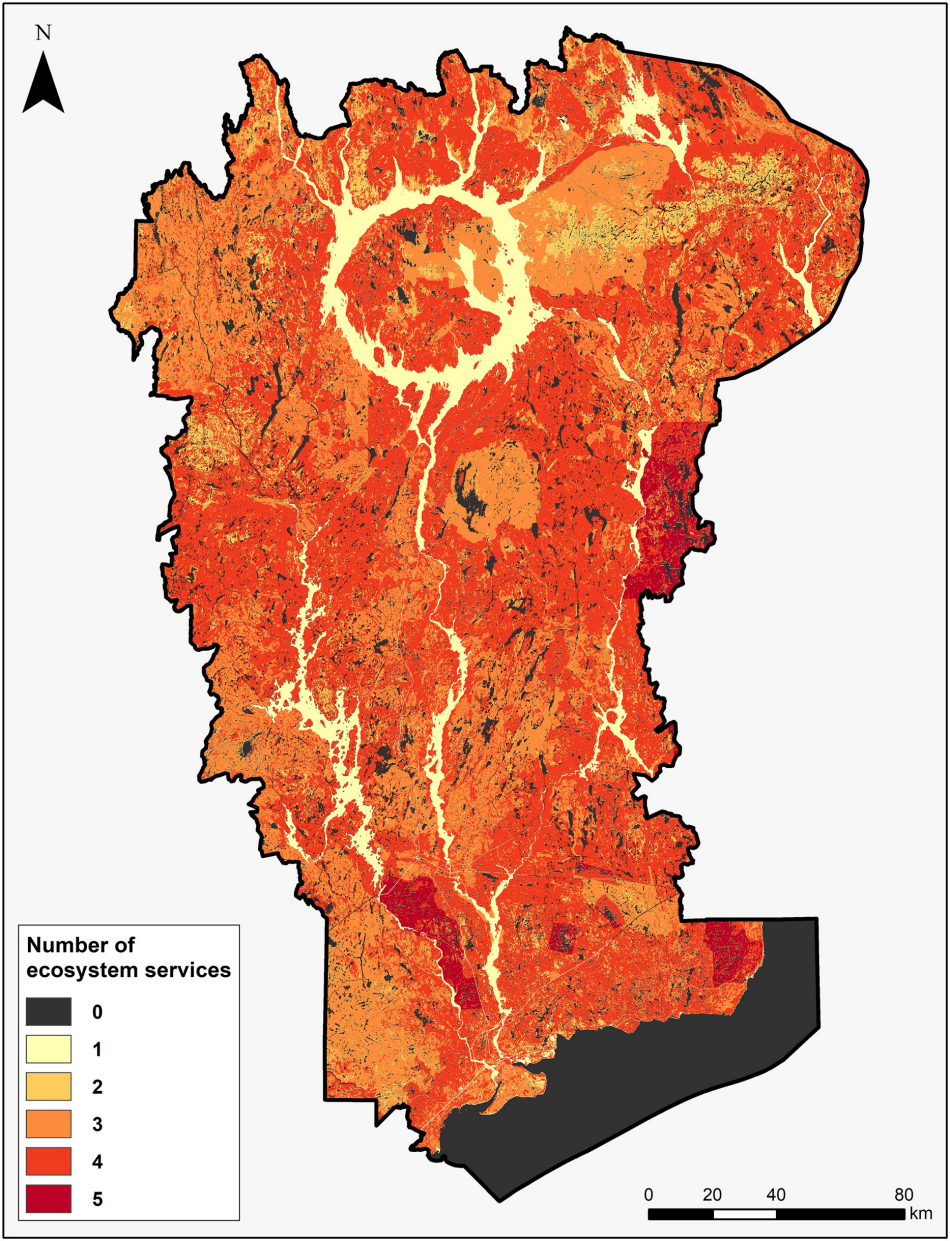
Location of areas that provide multiple ES in the MUWBR.

SPU areas are identified and estimated for each ES (Table 3; Fig 4). The entire area occupied by the reserve and, hence, all types of land uses are involved in the ES production (Fig 5).

**Table 3:** Agriculture in the MUWBR, surface area and economic value (source: Financière Agricole, [52]).

Culture	Surface (ha)	Production value (CAN$/ha/year)	Total value of the production (million CAN$ per year)
*Oats*	77	902	69.5
*Corn*	7	1206	8.4
*Cranberries*	29	2771	80.4
*Canola / Rapeseed*	6	3630	21.8
*Barley*	261	500	130.5
*Potatoes*	70	5111	357.8
*Prairie*	22	116	2552.0
*Vineyards*	24	3630	87.1
*Perennial crops and pastures*	876	116	145.4
*Undifferentiated Agriculture*	738	0	0
**Total**	**2 109**	**/**	**3452.9**

Timber is provided by forest areas which are not located in specific protected zones within the MUWBR such as biological refuges, ecological reserves or protected areas. These amount to a total potential production area of 3.33 Mha from broadleaf or mixed forest, coniferous forest, and cuts and regeneration zones (Fig 4). Food provision from agriculture is provided by only 2 019 ha in the MUWBR. Most of this land is planted under perennial crops and pastures (41,5%) and barley (12,4%), the two main agricultural uses, while 35% of this agriculture area is classified as undifferentiated crops (Table 4). Agriculture land uses are mainly concentrated around Baie-Comeau at the southern portion of the reserve which is the main of the MUWBR (Fig 3b). Freshwater for energy production is provided by the aquatic environments i.e. the broad water reservoir(s) spread over the entire territory of the reserve and representing approximately 300 000 ha (Fig 4).

**Table 4:** SPU surface and economic value for the key ES.

ES	SPU land use categories	Surface (Mha)	% of the RBMU	Economic value (million CAN$ per year)	% of the TEV
**Timber**	Forest under production	3.33	60,8	8.9	0.70
**Food from agriculture**	Agriculture area	0.002	5,5	3.5	0.27
**Freshwater for energy production**	Water reservoirs	0.3	0,04	572.0	44.87
**Global climate regulation**	Forest + Wetlands	4.03	73,5	688.6	54.00
**Habitat provision for biodiversity**	Habitat area of species with special status (e.g. rare, vulnerable, threatened species)	5.48	100	/	/
**Recreation and tourism**	Outfitters, controlled exploitation zones, wildlife reserves	0.20	3,6	2.0	0.16
**Cultural heritage and diversity**	Area of land claimed by the Innu Nation	5.48	100	/	/

Global climate regulation is provided by ecosystems having the capacity to store and sequester greenhouse gases. Here we consider only carbon storage and sequestration through live biomass of forests and wetlands which constitute a broad part of the MUWBR (74%) (Fig 4). SPU for the service of habitat provision for biodiversity is estimated for species with available data, i.e. plant species, avifauna and caribou species designated by the Act Respecting Threatened or Vulnerable Species in Quebec, the declination of the Canadian Species at Risk Act (http://www.atlas-oiseaux.qc.ca/index_fr.jsp). Sixteen designated species of birds have been recorded and observed in the reserve; however, hotspots can be identified (in the extreme west and east areas of the reserve and along a corridor that crosses the MUWBR from north to south, encompassing the Manicouagan reservoir). Secondly, three hotspots of designated plant species were also identified: the coastal habitat located in extreme south of the MUWBR which is linked to the bioclimatic domain of the white pine forest; a central area located midway between the Manicouagan reservoir and the coast, adjacent to the Manicouagan River; and the northeast sector, adjacent to the Manicouagan reservoir. Thirdly, MUWBR is located at the heart of the woodland caribou, *Rangifer tarandus caribou*, habitat which is a non-migratory designated species. Its historical habitat extends from the boreal forest to the northern United States, covering all the southern Quebec, but the recent population decline has resulted in a displacement of the southern limit of its habitat. The MUWBR now represents a third of this new habitat area for an estimated 2 200 to 3 100 woodland caribou.

MUWBR offers vast territories for recreational purposes (e.g. snowmobiling, fishing, hiking, camping) but our SPU for recreation and tourism was limited to outfitters, controlled exploitation zones and wildlife reserves where cultural activities are concentrated because of accessibility constraints (Fig 4). Finally, the entire territory of the MUWBR is considered as the SPU for cultural heritage and diversity because of the cultural services linked to indigenous traditional practices. First indigenous groups began to settle the territory of Manicouagan-Uapishka after the withdrawal of the last great glacier continental. This territory contains a large number of burial sites, gathering sites, hunting, fishing, trapping and gathering areas as well as travel routes that are claimed by the Innu community of Pessamit of which more than 2 000 members live in the reserve today.

An overlay map of the SPU is then produced and hilights hotspots providing from 1 to 5 key ES (Fig 5).

### 3.2. Economic valuation

Economic value of timber has been estimated from the management units of the Chief Forester Office and from the Tariff Zone values of the Wood Marketing Bureau. Wood volumes that can be harvested on a sustained basis over 150 years are first estimated and secondly valued based on the indexed grid of market values. Thus, the potential annual harvest value has been estimated at CAN$ 8,920,240. The net benefit of each agricultural production has been determined by the transfer benefit method based on three studies carried out in Québec [39,52–53] and adapted to local data. In these studies, net benefit is calculated on the basis of the difference between gross benefit (the market value of the agricultural products) and production costs (wages, machinery costs and different inputs needed for agricultural production). The total value of agricultural production in the MUWBR amounts to CAN$ 3,452,900 per year. Net benefits varied between CAN$ 116 and CAN$ 5,111 per hectare per year, depending on crop (Table 3). Freshwater for energy production is assessed from the profit generated by the 11 hydroelectric power stations which are located in the MUWBR. These power stations represent a total installed capacity of 8,545 MW, i.e. 18.2% of Hydro-Québec’s total electricity potential [54]. As the total profit generated by Hydro-Québec is CAN$ 3147 million, the profit generated at the MUWBR scale is estimated at CAN$ 572 million per year.

Global climate regulation is assessed for forests and wetlands separately for both carbon stock and sequestration, based on existing ecological studies of Québec. First, carbon stocks in forest is considered both for below ground carbon of trees and above ground carbon of trees. The former has been estimated at 120 tC/ha [55] and the latter has been estimated from 23.2 tC/ha for scattered hardwoods to 73.3 tC/ha for dense mixed forests [56] using LIDAR data. In the same way, carbon stocks of wetlands have been calculated for below ground biomass and above ground biomass [55–56] and estimated for the MUWBR at 234 MtC, i.e. 858 Mt CO2 eq. Secondly, we estimate carbon sequestration capacity for the forest and wetlands ecosystems. The sequestration capacity of forests has been evaluated at 0.1 tC/ha per year (i.e. 0.37 t CO2/ha per year) for the study area [57]. A carbon sequestration rate between 0.528 and 0.678 tC/ha per year has been estimated for a wetland located close to the MUWBR [58]. No other study allows us to obtain a representative sequestration rate for all the wetlands in the study area, as a result, this low estimate (0.528 tC/ha per year, i.e. 1.93 t CO2/ha per year) has been used for the economic valuation. The monetary values of the carbon stock and sequestration were then assessed using the social cost of carbon estimated at CAN$ 42.37 per t CO_2_ in Canada [59]. To take into account the temporal dimension of the service, this value was broken down over a 50-year period with a discount rate of 3% (i.e. recommended rate [59]). Thus, the economic value of carbon stock is estimated at CAN$ 445,221499 per year for forest and CAN$ 165,903, 520 per year for wetlands. The economic value of carbon sequestration is estimated at CAN$ 58,708,417 per year for forest and CAN$ 18,735,457 per year for wetlands. As a result, the total value for the global climate regulation in MUWBR is estimated at CAN$ 688,6 million per year.

Recreation and tourism ES were valued only for moose hunting which is the most practiced recreational activity within the MUWBR and for which data are available. Benefits derived from this activity have been estimatedbased on a consumer surplus equivalent to CAN$ 271.75 per hunter for a hunting season [60]. This value has been adapted to the MUWBR context by Doyon et al. [61] who estimated the annual value of moose hunting activities in the reserve at approximately CAN$ 2,000,000 per year.

By aggregating monetary use values of each of the five key ES, the total use value is calculated and estimated to be around CAN$ 1.3 billion per year. This value is mainly explained through freshwater for energy production and global climate regulation representing respectively 44.87% and 54.00% of the TEV.

## 4. Discussion

The ES framework is intended to be a tool for decision-making and for communication about the importance of ecosystems to sustain human wellbeing, focusing on derived economic and social benefits [32]. While the MAB Biosphere Reserves have fully integrated ES into their action plans, this case study illustrates the challenges and opportunities to complete an ES assessment at the scale of a specific biosphere reserve.

### 4.1. Selection of key ES

First of all, the need to select the key services on which the evaluation process will focus on, reveals the difficulty of conducting an exhaustive ES assessment of the territory. Indeed, other potentially significant and relevant ES are provided by the MUWBR (e.g. genetic resources, natural medicines, air quality regulation, erosion regulation, water purification, aesthetic values) but could not be evaluated due to lack of existing data. This challenge is not specific to this study: while a robust ES assessment requires full information regarding the different services provided by the ecosystems concerned, many studies must deal with a lack of data and also with a spatial and temporal heterogeneity of the available data when trying to assess these [62]. Authors of these study have adopted an adaptative scientific approach, based on academic expert discussion, in order to put priority on specific and relevant ES and, as a consequence, to complete the assessment and increase its effectiveness. This type of adapatative processes are highly recommended in post-normal science [63]. To go further in the support of decision management and improve the process of establishing priorities concerning ES assessment, the use of a deliberative process like the TRIAGE approach can be relevant when stakeholders are involved in the assessment process, which was not the case here. As a consequence, indigenous or local communities can have a weight in the process. TRIAGE helps to prioritize the assessment refining its aim and taking into account the potential changes in ecosystems and ES in the future [64].

### 4.2. Supply ES assessment

SPU mapping offers a relevant tool for decision-making as it leads to identify and locate the ES supply and thus provide a support for decision-making in a planning perspective. Moreover, SPU concept leads to opportunities for ES assessment by enhancing interdisciplinary collaboration between ecologists and economists. In the MUWBR, SPU for provisioning services (timber, food from agriculture, freshwater for energy production) are lands and water mass directly used by humans and, beyond the ecosystems naturally present within the reserve, are defined in terms of property rights, right of exploitation and accessibility. Regulating services (global climate regulation, habitat provision) are provided across the MUWBR while their benefits can be considered as regional or global. Cultural services are also provided in the all MUWBR but some of them are limited by accessibility (recreation and tourism). SPU mapping offers a synthetic vision of the ecosystems producing ES but should not make us forget that this ES supply varies in space, time and complexity, i.e. SPUs are dynamic. Indeed, complex key ecological processes are hidden like, for exemple, the symbiosis between trees and ectomycorrhizal fungi that contributes to the carbon storage and sequestration by boreal forest since the fungi receive energy from their host tree and, in return, deliver key nutrients such as nitrogen and phosphorus [65]. Regarding geographical and temporal variation, it is relevant to have in mind that the rates of storage and sequestration can vary over the lifespan of a tree, i.e. usually high rates of sequestration (low storage) when trees are young, and slowing sequestration but high storage when trees are older. In the same way, the value of for food from agriculture in SPUs are reduced when some fields are left fallow. It would also be relevant, as advised by Andersson et al. [66] to complement this SPUs approach with elements that concern their ecological condition, the quantity and quality of ES they provide, and the ES benefiting area they target (see also Burkhard et al. [42]. Finally, corridors that connect SPUs are important for the ES supply and should be an important consideration in planning and management [67]. For example recreation provided by moose hunting depend on moose habitat, which may be determined by not only the SPU, but also on the connectivity to other sites and the larger region.

### 4.3. Demand ES assessment

Monetary valuation offers a formalized framework to assess the economic benefits derived from ecosystems in a tangible metric for decision-makers. However, authors are aware of the non-exhaustivity of monetary approaches to account for ES demand and the multi-dimensional nature of human well-being and as Martin and Mazzota [68], they expect to see more ES assessments based on non-monetary valuation. The latter allows to articulate plural values through different qualitative and quantitative measures other than money [69]. Although the major international initiatives and programs (MA, TEEB, IPBES) have acknowledged the the need to conduct this type of valuation, it does not yet constitute a formalized methodological framework and is still difficult to operationalize [70–71]. Monetary values are still powerfull for decision-making since they are easier to aggregate in order to obtain a total economic value but they do not allow to highlight the trade-offs between ES involving multiple interests [72]. In this study, monetary valuation focused only on use values as non-use valuation methods based on revealed preference approaches are expensive, time-consuming and need good quality and large data set to outweigh the possible market imperfections and policy failures which can distort the estimated ES value [50]. Authors agree with Plottu and Plottu [64] saying that non-use values stem from more different levels of choice problems than use values and thus, must be apprehended in a multidimensional framework. In the MUWBR, 99% of the total use value (5 ES) is explained through freshwater for energy production and global climate regulation.

Mainly influences by the land use, the total use value is also influences by specific contexts i.e. climate change and the scarcity of water resource which contribute to increase the ES value. A future evolution of these land uses and/or context would reveal new key ES and lead to a change in the distribution of the total use value, which raises some question regarding the implications in terms of ecosystem management.

For the value estimate, a suitable pricing method was chosen for each of the ES indicators based for a majority of them on previous ES valuations. In this study, because provisioning services are market goods, their economic values have been estimated through market prices (timber) and production function (food from agriculture; freshwater for energy production). Regulating ES (global climate regulation) has been estimated considering the social cost of carbon while cultural ES (recreation and tourism) has been valuated through production function. Because these use values are based on market prices, they provide some insight concerning the ES demand. However, because they focused only on one indicator for each ES flow, they don’t provide a full assessment of the ES demand as they don’t provide information about the consumption, non-satiated or conservation demands [42,73]. For example, the economic value of moose hunting doesn’t consider the potential social value of this activity or the possible conflicts with the moose conservation demand. Habitat provision and cultural heritage and identity were the two ES that couldn’t be assessed because no relevant economic indicators have been identified. Even if they are key ES for the MUWBR, they have no weight in the total value because their assessment couldn’t be undertaken, even though cultural values are of particular importance to the Innu community of Pessamit. As a result, the approach favors specific ES for which monetary indicators exist. However, the authors are convinced that the monetary values they highlighted can be used as a strong argument for not undervaluing the value of preserved ecosystems compared to exploited ecosystems. To go further and improve the assessment, a deeper use of multicriteria analyzes of ES based on ecological, economic and social considerations, considering a pluralism of values can be relevant [74]. Multicriteria analysis makes possible the formal integration of multiple values after each of them has been assigned a relative weight [75]. The output of multicriteria analysis is a ranking of values that serve as a support for decision and management without the need to convert all values to a single unit. For example, the current status of the caribou stock and the importance of the MUWBR as a habitat for this species could be considered in the light of ecological (e.g. stock change) and social (e.g. efforts made to conserve the species) values. Another way to improve the assessment can be the use of the approach of ES bundles which is increasingly recommended for ES assessment. ES bundles are defined as sets of ES that repeatedly appear together across space or time and are used to analyze interactions among ES [76]. The approach is thus a useful tool for improving the management of ecosystem and identifying common ES tradeoffs and synergies: trade-offs arise when the provision of one service is enhanced at the cost of reducing the provision of another service, and synergies arise when multiple services are enhanced simultaneously [76]. However, if the use of multicriteria analysis and ES bundle approach can be some opportunities, it is also a challenge as it requires a deep understanding of the ES and their interrelations, accounts for the complexity and can be more difficult to be accounted in decision-making processes.

### 4.4. Biosphere reserves

ES assessment can help a manager or a decision-maker to make a choice based on the importance of the different ecosystems and the societal dependencies on them. Taking this ES concept into account marks the evolution of environmental management in general and the evolution of strategies for Biosphere Reserves to justify the conservation of biodiversity. The ES approach is characterized by a normative ambition: defining, categorizing and valuing ES in order to improve the protection of biodiversity. However, the objective of these ES assessments in MAB Biosphere Reserves seemed not clearly defined. Is it to convince with new tools of the need to better protect biodiversity? Is it to attract more funding for conservation? Is it to monetize some components so far remained outside the market economy and bring it to a new market? Based solely on the desire to establish an initial state of the situation, there is a risk that further assessments will evolve towards more robust methods which will not allow comparison with this initial ES assessment. Identifying and collecting the data for standardized indicators through time is cautious so that even if the approaches change, ES assessment will be based on a standard set of data and baselines would be re-establish using comparable methods. Finally, one can imagine that this concept will lead to a shift of the main objectives pursued by protected areas or the creation of new types of protected area, entirely devoted to the protection of ES [5,77–78].

## 5. Conclusion

ES assessments are carried out worlwide from local to global scales taking into account the dependencies between society and nature in order to promote biodiversity conservation. In this context, MAB program of UNESCO has initiated local ES assessments within its world network of Biosphere Reserves. Despite their common use, some challenges remain mainly based on the lack of data and a validated analytical framework. The assessment conducted within the Manicouaga-Uapishka World Biosphere Reserve highlights some of these challenges but also some opportunities to overcome them. First, it is difficult to conduct an exhaustive assessment of the ES provided by the territory as they can be very numerous (e.g. MA classification, CICES list) but a set of key services can be defined using a strategic approach based on specific criteria including the presence of available data. Second, the ES supply is difficult to estimate in common metrics since it requires significant local ecological knowledge and refers to different components of the ecosystem (e.g. stock of resources, ecological process, landscape structure) but the concept of SPU can be an alternative to this estimate using a cartographic approach to spatialize supply and quantify the area of the involved territory. Third, the TEV concept remains the privileged tool for conducting ES assessments. However, it requires the identification of robust monetary indicators and available data. Moreover, TEV does not reflect all the ES of the territory since only the use values of the key ES have been aggregated. Use-value leads to estimate only provisioning ES because of the direct use of ecosystem, recreative ES because of the indirect use of ecosystem and some of regulating ES for which costs have been calculated and validated because of their societal importance (e.g. global climate regulation). Despite its non-completeness, this value serves as a basis for discussion in decision-making processes and generally argues for the sustainable use of resources. Defining a clear objective for ES assessment within biosphere reserves is an essential point to guide the assessment process. Used as an initial state of the ES, one must be aware that futures assessments will not necessarily be comparable with current assessment because of the current methodological and conceptual scientific developments. Nevertheless, the latters are widely encouraged to be as comprehensive as possible but must be considered with the operationality of the tools.
